# Unequal Access to Transcranial Magnetic Stimulation and Electroconvulsive Therapy for Treatment-Resistant Depression in the United States: Advancements in Therapies and Continued Disparities

**DOI:** 10.31083/AP47427

**Published:** 2026-10-12

**Authors:** Charles Lai, Peter Bota, Jonathan Steve Shaw

**Affiliations:** ^1^School of Medicine, California University of Science and Medicine, Colton, CA 92324, USA; ^2^Research & Development, Brain Health Solutions, Costa Mesa, CA 92626, USA

**Keywords:** electroconvulsive therapy, transcranial magnetic stimulation, bioethics, psychiatry, depressive disorder, treatment-resistant

In September 2022, the United States (US) Food and Drug Administration (FDA) approved the use of the Stanford Accelerated Intelligent Neuromodulation Therapy (SAINT) protocol for treatment-resistant depression [1,2]. The SAINT protocol is the first commercially available accelerated neuromodulation protocol and among the first neuromodulation techniques to use neuroimaging for the treatment of mental illness [1]. Its FDA approval indicated rapid progress in psychiatric neuromodulation, but ethical concerns pertaining to treatment accessibility remain [3].

The SAINT protocol involves patients undergoing both structural MRI and resting-state fMRI for personalized targeting for stimulation of the left dorsolateral prefrontal cortex (LDLPFC) to the subgenual anterior cingulate cortex circuit using multiple high-dose, intermittent theta-burst stimulation over a 5-day treatment protocol [2,4]. A small clinical trial found the SAINT protocol to be more effective than sham stimulation in alleviating treatment-resistant depression [4].

In terms of beneficence and nonmaleficence, careful consideration of individual patients and conditions is necessary for the selection of an optimal neuromodulation technique for a treatment-resistant psychiatric disorder. However, the lack of equitable access to many neuromodulation techniques, including transcranial magnetic stimulation (TMS), poses ethical concerns [3]. Compared to most psychopharmacological options, TMS is a high-cost treatment with limited insurance coverage and, therefore often inaccessible to patients outside of affluent regions or higher socioeconomic classes [3,5]. Between 2014 and 2020, only 5.13% of facilities in the US provided electroconvulsive therapy (ECT), ketamine infusion therapy, or TMS; neuromodulation access increased slightly from 0.18 to 0.24 per 100,000 US adults over this time period [6]. Recent literature, albeit complicated by limited data due to inconsistent reporting, has suggested that the increased availability of TMS in the United States is driven by an increase in privately-owned TMS practices [3,6].

This is concerning with regard to equity, as approximately 23.0% of adults living in nonmetropolitan areas in the US have been diagnosed with a mental illness [7]. The median drive time to mental health facilities was 18.5 minutes in large rural areas and 26.2 minutes in small or isolated rural areas, and only 13.2 minutes in urban areas [7]. This distance issue is exacerbated by the nature of conventional TMS protocols, which target the LDLPFC 5 days a week for 6 weeks [2,4]. These frequent appointments can pose significant barriers to access for low-socioeconomic-status patients, especially those living in rural areas who may need to drive significant distances to access care. Additionally, insurance companies have created additional barriers by generally requiring documented failure of therapy and multiple pharmaceutical treatments prior to approving TMS [3,5].

The 5-day timeline of the SAINT protocol theoretically addresses certain logistical concerns, but the required resting period of at least 45 minutes between individual treatment sessions during those 5 days precludes patients from working during normal work hours while undergoing the SAINT protocol [4]. Additionally, since the SAINT protocol has only been FDA-approved for a few years, its coverage by insurance companies is limited. The SAINT protocol is also highly resource-intensive as it requires structural and functional MRI, making standard ECT potentially more cost-effective in the US and internationally.

ECT is a well-established and effective neuromodulation technique for moderate-to-severe major depression, acute mania, catatonia, and severe affective disorders when standard pharmacological agents cannot be used or are ineffective [8]. ECT providers in the US reported barriers to expanding existing ECT services, including a lack of physical space, stigma on the part of patients, transportation problems, and a lack of administrative support [9]. Compared with their university-affiliated colleagues, community hospital ECT practitioners are confronted with an additional barrier of limited knowledge among potential referrers, underscoring disparities in healthcare access between rural areas and urban centers with teaching hospitals [7,9]. Additionally, unlike TMS, ECT requires anesthesiologists, whose competing clinical responsibilities further complicate the running of ECT services [9].

In the US, the availability of ECT also varies by region; New England has the highest average per capita number of ECT providers of 6.4, and the West South Central states have the lowest average at 1.1 [9]. This geographic variation and the need to travel to specialized centers limit ECT access much like TMS access, however, legal issues increasingly represent a more significant barrier to ECT access across US states and territories.

In the US, laws and regulations concerning ECT appear to be becoming more restrictive over time [8,10,11]. In 2006, there were 33 geographical jurisdictions in the US where state laws and administrative codes did not comment on the use of ECT [8]. At that time, ECT regulations generally had a minimal goal of specific consent, though certain states, such as California and Texas, enacted laws governing nearly every aspect of the procedure [8]. By 2018, there were only 6 states with no laws regarding ECT, and by 2025, Georgia was the only state without state laws regulating ECT, though proposed bills are under legislative review [10,11]. This is a worrying trend, especially as some state laws were found to be using long-disproven language such as, “there is a division of opinion about the effectiveness” of ECT [11]. Several states and jurisdictions (California, District of Columbia, Florida, Nevada, Oregon, Texas, Washington, and Wisconsin) have also included ECT among those medical procedures (psychosurgery, induced abortion, sterilization, and experimental treatment) classified as “extraordinary treatments” that cannot be performed on patients lacking capacity, even in emergencies [11]. Variability in geographic availability and legal regulations around involuntary ECT further restrict access, as requirements like additional court paperwork increase psychiatrist workloads and delay treatment [12].

Overall, despite advances in accelerated TMS protocols that theoretically address some of the barriers to TMS access, the high cost of TMS will continue to limit its accessibility in under-resourced populations in the US. Additionally, the increase in restrictive and often poorly-researched state regulations regarding ECT is a concerning trend which psychiatrists in the US should raise with their local and state government officials.

## Abbreviations

FDA, United States (US) Food and Drug Administration; SAINT, Stanford Accelerated Intelligent Neuromodulation Therapy; TMS, transcranial magnetic stimulation; ECT, electroconvulsive therapy.
